# Dental Advertising—Versus—Dental Ethics

**Published:** 1859-07

**Authors:** C. C. Dills


### DENTAL ADVERTISING—VERSUS—DENTAL ETHICS.

BY C. C. DILLS.

Reply to "Dental Advertisements"—by Dr. J. Richardson.

THAT dental writers are lamentably *theoretical* is a fact acquiesed in by all:—and while this state of things exists only with reference to matters arbitrary, or wherein no violence is done to principle, or *ethics*—lookers-on are excusable for non-interference:—otherwise they are not.

"The dental profession is in its infancy," (*Slosson*) and prominent among matters claiming its attention is its *ethics*. This fact has not been disregarded, while strict ETHICAL PRINCIPLES may have been, in a too whole-sale treatment of the subject—or by the "slip of a too hurried pen"—*or* by one wielded by hands not wholly unsullied by efforts to accomplish *private ends*, under so soul inspiring a garb.

"Many times and oft" we have thought some modern views of dental ethics *too progressive*, if not *absolute conflictions* with moral rights possessed by every man—and with which *ethics* should ever harmonize. Yet we have been deterred from remonstrance, either "from feelings of delicacy towards the profession," *or* from a knowledge of the *fact* that even flagrant errors, adduced in behalf of *ethics* are generally supported, on the one hand, because they are hoped to err in the *right direction:*—and on the other, because to oppose them would be to run imminent risk of receiving at the hands of some "occupying high stations in the profession" "a tremendous letting" *down* by a *simple twist* of *their* almighty wrist.

We find several of these objectionable views conveniently *compiled* for us, in a recent article on "Dental Advertisments by J Richardson," and will have the *fool hardiness* to combat them;—asking the sympathy (in view of the *animal* force against us) of the profession—*only*, as, *by legitimate deductions* we prove said compiler's pen to have been "too hurried"—"or wielded by hands not wholly unsullied," &c.—and backed by a mind incapable of laying "aside every weight and the sin which doth so easily beset" it, and pressing forward to the possession of truth, equity and right *only*. It is the "judgment" of Professor Richardson that, "Dental advertising should be limited to a simple announcement of the practitioners name, title, and place of business;"—all of which *sounds very well*—we might say *desirable*—aye that "it is a consummation devoutly to be wished:"—what then? is it *therefore* correct, practical—*utilitarian!* and shall it *now*—and the *world over alike*—be made "a standard of legitimacy!" (*And especially before* the legitimation of the matter—and while it is unheeded on all hands—shall it be considered in harmony with dental ethics—(we make no appeal to moral principle) that the offender (?) be—*indiscriminately*—save as maliciousness may dictate—"*summarially* read out" of the profession,) We think not, *many* think not, and nine tenths of the profession utterly ignore any such would-be rights on the part of a profession, to the usurpation of ones own prerogative and inherent moral right, to manage his own peculiar affairs,

But let us pass to more significant facts and deductions, and inquire—*what are ones undeniable rights*, moral and political;—and make our deductions from them with reference to "Dental Advertising" *viz Ethics*,

"We hold these truths to be self-evident, that all men are created equal; that they are endowed by their Creator with certain inalienable rights: that among these, are life, liberty, and the pursuit of happiness."

It is likewise "self evident" that in "the pursuit of happiness" is recognized the right of *vocation*, with subordinate rights—to be established by municipal governments, and among which is that of *advertising* said vocation. Now while unlimited license is *taken* in advertising, it is yet an easy matter to *define limits—within which we may act and claim immunity from the "judgment" of any man—or set of men—short of* "in Congress assembled;"—(*And further—for any man to advance as his "judgment,,, views so striking at the foundation of these "inalienable rights" as are those of J. Richardson's—and "presto" would legitimize them*—IS ARROGANCE CONSUMMATE, PITIABLE!—"Let no such man be trusted"—*with handling Dentel ethics.*

The business world has defined these limits, and so self-evidently in harmony with both *rights* and *ethics*—that we give them, *as sanctioned by the late "Ohio Editorial Convention"*—without comment.

"An advertisement should set fourth fully the capacities of a business, or, in other words, should present in detail the particular inducements offered in selling them, their quality—style, prices &c, In short he must embody in his advertisment *just what he would say to an individual, were he to visit him to solicit his patronage. An advertisment should be plain—concise—and truthful.*"

*Herein—gentlemen* of the profession do I stand—*and act:*—ready by *you* to be judged, and pay the penalty (?) of my doings!—

> "*Yet* such extenuation let me beg,
> As, in reproof of many tales devised,
> By smiling Pick—thanks and base Newsmongers,
> I may, for some things true, wherein my youth
> Hath faulty wander'd and irregular,
> Find pardon on my true submission."

Again, as "Calumny, though known to be such, generally leaves a stain on the reputation:"—I would remonstrate that "All are not thieves that dogs bark at."

Nothing is more apparent than the feeling prompting "Dental Quacks No. 3, by H. R. Smith" and "Dental Advertisements by J. Richardson," when considered in relation to our article on "Dental Quacks"—and the relation in which these gentleman stand one to the other. To these gentlemen would we extend the hand of congratulation, that "the mark of the prize of their high calling" is *so high:* and that upon its attainment, there is in store for them a profession's love and gratitude—inasmuch as they have *saved it*, and purged it of all sin and uncleanness," by "*a process* of *active medication*,,

We had desired to notice other *judgments* of these "fast gentlemen," "but space at present forbids:" before being "read out" *loud* however we may have occasion to refer to them again;—in the meantime we will pursue the even tenor of our way—advertising to our patrons very much after the manner we would talk to them—*or the profession:* and where we can only say a *few words*—will *exhort* them—"for Heaven's sake" "see small bills:" (of which we are just now having some struck off, and will *send out*, of course: hoping for them as wide circulation, as has favored our former advertisments.)

